# Editorial

**DOI:** 10.1120/jacmp.v8i1.2656

**Published:** 2007-02-27

I am sure you have noticed the new look for the JACMP. We have completed a migration to the latest version of the Public Knowledge Project Open Journal Systems software. This robust and flexible tool is available to anyone with web access without cost. The Public Knowledge Project is an initiative of the University of British Columbia and is funded by the Canadian Federal Government. Competing commercial packages cost many thousands of dollars to initiate and support and may also charge a fee per article processed. It is unlikely the JACMP could offer articles without cost if the Public Knowledge Project did not exist. Already the new software is making a big difference in the way we handle our submissions. Hopefully we will be able to handle and process our articles with even greater efficiency and security. Many of you are aware that the JACMP offers, to my knowledge, the only double‐blind peer review model in medical physics.

In this issue, we wish to say goodbye and to two good friends: Janelle Molloy and John Horton. Both served as Section Editors for Radiation Oncology, and later, John served as Editorials Editor. To both of you, I offer many thanks for your years of service to the JACMP.

Michael D. Mills, PhD

**Table:** 

**The Journal of Applied Clinical Medical Physics**	
**Editorial Board 2007**	
Editor‐in‐Chief	Michael Mills
Deputy Editor‐in‐Chief	Timothy Solberg
**Associate Editors**	
Radiation Oncology Physics	Nzhde Agazaryan, Salahuddin Ahmad
	John Antolak, Ivan Brezovich
	Jefferson Fairbanks, B. Gino Fallone
	Benjamin Nelms, Matthew Podgorsak
	Nikos Papanikolaou, William Salter
	Mehrdad Sarfaraz, Lu Wang
	Albert Zacarias
Medical Imaging	Geoffrey Clarke, William Pavlicek
Radiation Measurements	Larry DeWerd
Radiation Protection & Regulations	James Rodgers
Nonionizing Topics	Bhudatt Paliwal
Other Topics	Timothy Solberg
Book/Media Reviews	~
Editorials	~
**Journal Production Team**	
Proofreader	Heather Shand
Copy Editor	Ann Fothergill‐Brown
Layout Editor	Laura Shand
Manuscript Manager	Bonnie Ratcliff

